# *LRRTM4* Terminal Exon Duplicated in Family with Tourette Syndrome, Autism and ADHD

**DOI:** 10.3390/genes13010066

**Published:** 2021-12-27

**Authors:** Raymond A. Clarke, Valsamma Eapen

**Affiliations:** Ingham Institute, School of Psychiatry, University of New South Wales Sydney, Sydney, NSW 2170, Australia; v.eapen@unsw.edu.au

**Keywords:** NTSC, gender bias, neurexin, ADHD, OCD, ASD, CNV

## Abstract

Tourette syndrome (TS) is a neurodevelopmental disorder characterised by motor and vocal tics and strong association with autistic deficits, obsessive–compulsive disorder (OCD) and attention-deficit/hyperactivity disorder (ADHD). The genetic overlap between TS and autism spectrum disorder (ASD) includes those genes that encode the neurexin trans-synaptic connexus (NTSC) inclusive of the presynaptic neurexins (NRXNs) and postsynaptic neuroligins (NLGNs), cerebellin precursors (CBLNs in complex with the glutamate ionotropic receptor deltas (GRIDs)) and the leucine-rich repeat transmembrane proteins (LRRTMs). In this study, we report the first evidence of a TS and ASD association with yet another NTSC gene family member, namely *LRRTM4*. Duplication of the terminal exon of *LRRTM4* was found in two females with TS from the same family (mother and daughter) in association with autistic traits and ASD.

## 1. Introduction

Tourette syndrome (TS) is a neurodevelopmental disorder characterised by motor and vocal tics, a prepubertal age of onset, a waxing and waning course and improvement of symptoms in adulthood [1,2]. Autistic features, obsessive–compulsive disorder (OCD) and attention-deficit/hyperactivity disorder (ADHD) are commonly associated with TS [1,3]. TS has high degrees of genetic inheritance, and the familial nature of TS has been evident since the time of its original description by Gilles de la Tourette in 1885. Twin studies suggest a monozygotic to dizygotic concordance of 77 to 23%, and family studies have consistently shown a 10- to 100-fold increase in the rates of TS in first-degree relatives [4]. 

Phenomenological, epidemiological and pathogenetic evidence all indicate a strong overlap between TS and autism spectrum disorder (ASD) [3,5]. TS and ASD both begin during childhood and mostly affect males. Clinically, symptoms such as obsessions, compulsive behaviours, involuntary movements (tics in TS and stereotypies in ASD), poor speech control and echolalia are common in both conditions [2]. Genetic epidemiology studies also indicate common susceptibility genes in both disorders. Furthermore, the rate of autism in TS is higher than that expected by chance, with ASD present in ~22% of children and over 8% of adults [6], whereas autistic symptoms occur in a third of TS cases and a further two-thirds show social deficits related to the autism spectrum [3].

**Figure 1:**
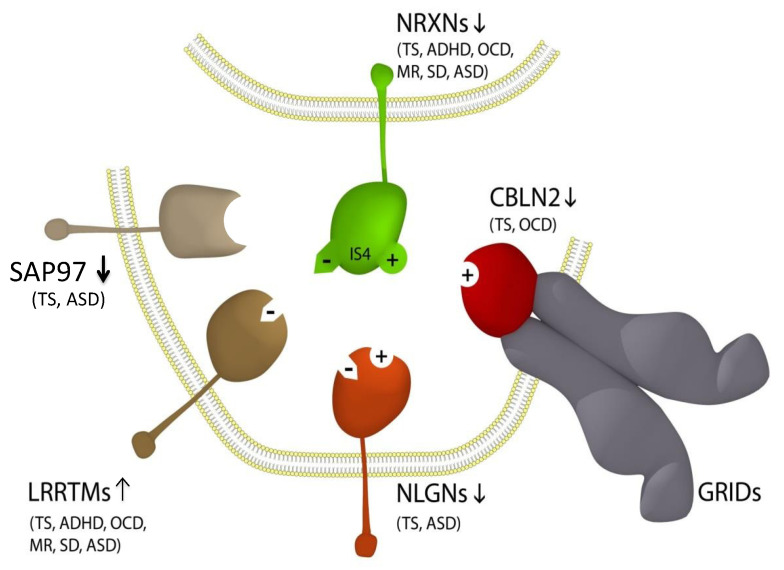
Updated NTSC pathogenetic model for Tourette syndrome (TS) and ASD [7] implicates the full complement of neurexins (*NRXN1-4*) and the principal neurexin trans-synaptic cell adhesion ligand gene families through multiple means of association: neuroligins (NLGNs), leucine-rich repeat transmembrane proteins (LRRTMs) [14], cerebellin precursors (CBLNs) and synapse-associated proteins (SAPs/DLGs) [7]. Presynaptic *NRXN1-3* form competitive trans-synaptic complexes with postsynaptic ligands NLGNs, LRRTMs and CBLNs in the formation and/or maintenance of neuronal circuitry within the brain. GRID1, which complexes with the CBLNs, has been deleted in ASD [15]. Vertical arrows indicate putative pathogenic dose effects. *NRXN1* isoforms with (+) and without (−) the 30-amino acid insert at splice site 4 (IS4), which indicates competitive binding of *NRXN1* between its ligands. *NRXN4*/*CNTNAP2* bind across the synapse with *SAP97*/*DLG1* [16,17]. Comorbidities listed are those associated with the TS translocations and copy number variations (CNVs) affecting the respective genes [7,14,16,18,19,20,21,22,23,24,25]. Note that this list of associations is by no means exhaustive, with new NRXN mutations identified regularly.

Despite the identification of candidate genes in TS and ASD, there is limited understanding of the exact pathogenetic processes in either TS or ASD. However, an association with the neurexin trans-synaptic connexus (NTSC), which regulates neuronal cell adhesion and neurotransmission, has been proposed to link TS and ASD [7]. The NTSC includes the full complement of presynaptic neurexins (*NRXN1-3*) and their postsynaptic ligands the neuroligins (NLGN1-3), cerebellin precursors (CBLNs with their GRID1 complexes) and leucine-rich repeat transmembrane proteins (LRRTMs), all of which have been implicated in TS and ASD through multiple associations (Figure 1) [7]. The prime example of this association is the large number of mutations of the *NRXNs* in TS and ASD and more especially in the *NRXN1* gene. *NRXN1* has also been implicated in ASD through haplotype sharing [8], as have other ASD and TS risk genes, including the *IMMP2L* gene, which has also shown repeat linkage to and haplotype sharing with ASD in multiple populations [8,9,10,11,12,13]. In this study, we report TS and ASD in association with yet another NTSC gene family member, namely *LRRTM4.*

## 2. Materials and Methods

Copy number variation (CNV) analyses were performed on blood samples from the proband and her family using two different microarray CNV detection platforms: the Agilent SurePrint G3 Human Whole Genome Microarray 2 × 400 K (60-mer oligo probes; mean effective resolution: 0.06Mb) and the Illumina CYTOSNP12 vs2.1.

HPLC was used for amino acid profiling and gas chromatography and mass spectrophotometry for organic acid profiling of a blood sample from the proband. The study has been approved by the Human Research Ethics Committee of the Sydney South West Area Health Service (Project id. 08/CRGH/233).

## 3. Results

### 3.1. Clinical Assessment

The proband was a 9-year-old female referred by her mother following a history of behavioural, emotional and sensory concerns. A comprehensive assessment revealed other concerns, including restricted interests, repetitive behaviours, social difficulties and anxiety. Further assessment confirmed a diagnosis of autism spectrum disorder (ASD). It also became evident that in addition to the restricted repetitive behaviours that are characteristic of ASD, the patient also exhibited finger and body movements as well as animal sounds such as barking, meowing, etc. The frequency and intensity of these behaviours were noted to increase following the commencement of stimulant medication for her ADHD symptoms. This prompted further assessment by a child psychiatrist when it became evident that the patient had tics as early as her preschool years, including a nose twitch at 2 years of age. Since then, over the years, the proband has had a variety of motor tics, including blinking; eye staring; nasal twitching; flaring of nostrils; kissing self; gripping lips and other lip movements; facial grimacing; head nodding forward, backward and sideways; moving hair out of eye tic; fingers drumming, tapping, flexing and touching; abdominal contractions; body wiggling; leg flexing, extending and kicking; adjusting, picking and chewing clothes; picking skin and nails; poking self; chin on chest posture; stroking, especially her legs; complex movements such as stomping and skipping; abnormal gait in the form of raising the leg high with each step, etc. She has also exhibited a number of vocal tics, including sniffing; throat clearing; coughing; “ugh” and other baby sounds; t-t-t sounds with coordinated hand movements; squeaking, shrieking, screaming and other high-pitched sounds; barking, growling and other animal sounds such as chickens, cats, gorillas, etc. 

Other tic-related behaviours include echolalia (repeating words such as “pineapple” or “monkey” or phrases such as “Rum... Rum... I am in Mummy’s car”), echopraxia (copying and imitating, e.g., jumping onto the doctor’s consulting chair and saying, “I’m Dr K”) and non-obscene socially inappropriate (NOSI) behaviours, such as saying “you are ugly” and then laughing inappropriately. She also reported having a number of obsessive–compulsive behaviours that fall into the tic spectrum of behaviours. For example, she had to do things in a particular way to get it “just right”, with pencils facing one way, colours not to be mixed and shoes and other things needing to be in a particular place. She also had an obsession with LEGO® and miniature items. She also exhibited self-injurious behaviours such as picking her nail or skin until they bled. She also had emotion regulation issues and rage attacks, with aggression mainly directed towards her brother, which have been a significant issue in the past but are reported to be better now. She has always been a poor sleeper. Anxiety is reported to make her tics worse. She reported to be able to suppress her tics voluntarily for a period of time, such as generally staying “under the radar” at school and coming home to “release” the tics. On certain days when she was not able to suppress the tics or cope effectively, her mum would get a call asking for her to be picked up from school. 

**Figure 2:**
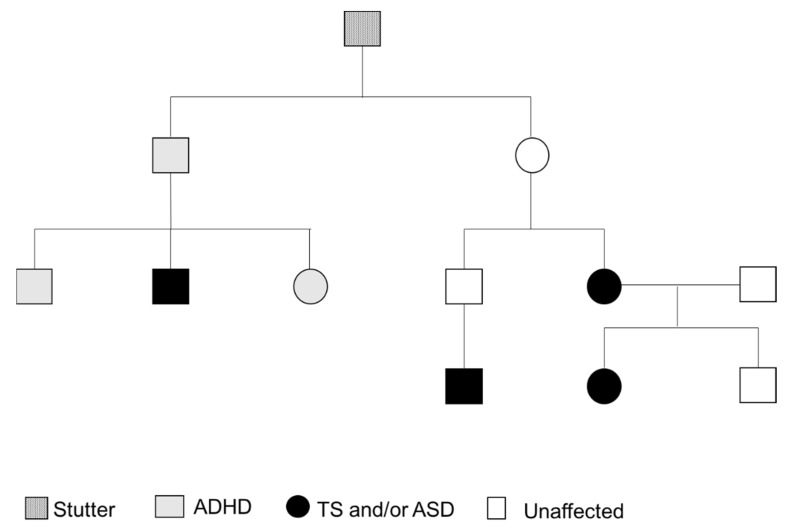
Family Pedigree.

Her early developmental history included myoclonic and febrile seizures at 18 months. She had a stutter, her speech was delayed and there was also some slight delay in other developmental milestones. It is worth mentioning here that there is a family history of TS, autism, stuttering and ADHD (Figure 2). It appears that the proband had significant ear infections, but things became better after treatment with antibiotics. There were some social difficulties in terms of making friends and she would often play alone and not engage in group games. However, she joined in if she knew the other kids or if they were doing something of interest to her. Currently, she sits with other kids opportunistically rather than being consistently part of a group. She has a number of sensory features, such as perceiving noises as too loud (e.g., in the classroom), not eating certain foods and, in particular, having issues with certain textures of food items. She can read other people’s facial expressions well but she may occasionally show facial expressions that are inappropriate for the context, and cuddles or hugs are on her terms. 

Regarding her family history, her mum reported having had nasal flare movements and other tics, and the maternal uncle’s son has been diagnosed with Tourette syndrome. Another maternal uncle is reported to have had ADHD, and he has one son with ADHD and autism and a daughter with ADHD (Figure 2). The maternal grandfather and his brother had stutters and other mental health difficulties, although not diagnosed. 

In summary, the female proband was diagnosed with Tourette syndrome with multiple motor tics and one or more vocal tics that have been present for more than a year and characterised by a waxing and waning course and one type of tic being replaced by another. The proband also has tic-related obsessive–compulsive behaviours and self-injurious behaviours, but she did not meet the diagnostic criteria for ADHD. The mixed features of autism spectrum disorder and the tic spectrum of behaviours is consistent with a family history of Tourette syndrome in association with autism. 

### 3.2. Genetic Analysis

**Figure 3:**
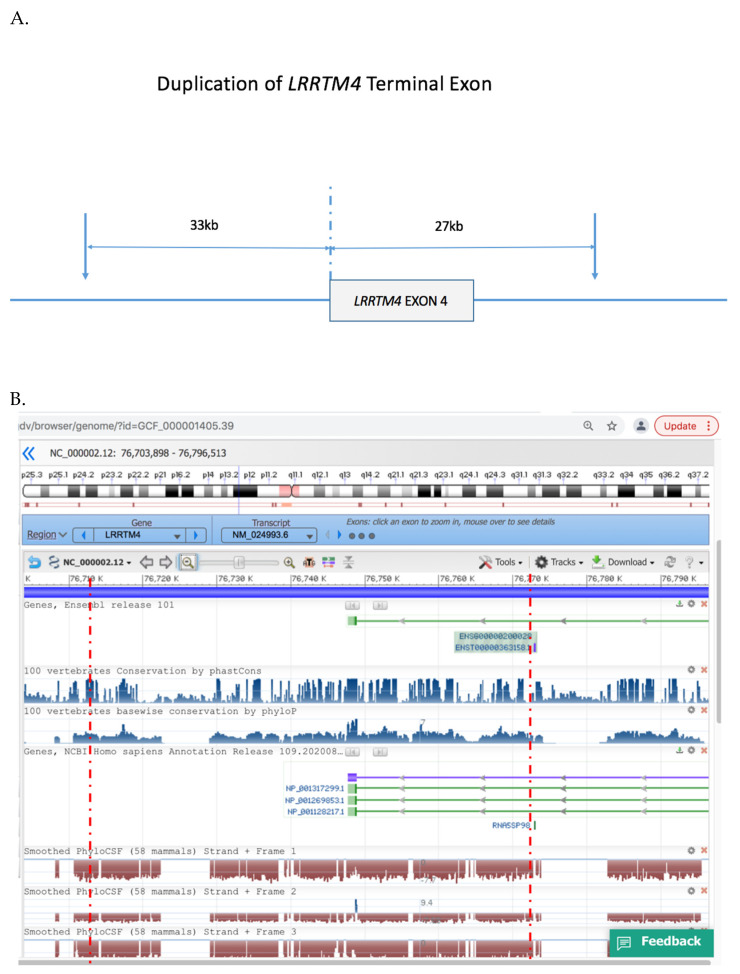
Duplicated region of *LRRTM4* gene. (**A**). Schematic of *LRRTM4* terminal exon duplication, (**B**). NCBI profile of the genomic region duplicated in the proband and her affected mother, inclusive of the terminal exon of *LRRTM4* inclusive of conserved noncoding regions. Red broken lines indicate the minimal critical region of the duplication. Reproduced with permission from NCBI Genome Data Viewer.

We performed microarray analysis of DNA blood samples from the family. We corroborated results by the use of two different CNV detection platforms (the Agilent SurePrint G3 Human Whole Genome Microarray 2 × 400 K platform and the Illumina CYTOSNP12 vs2.1 platform). Both platforms detected a heterozygous duplication of the terminal exon of *LRRTM4* in the female proband and her affected mother (Figure 3) which was absent from her unaffected father and brother. The minimal size of the duplication was ~60 kb arr 2p12 (76,941,049–77,001,017) and spanned the terminal exon of *LRRTM4* and extended ~33 kb 3′ of the gene. No other CNVs were evident in the proband or her mother.

### 3.3. Biochemical Analyses 

Amino acid profiling by HPLC indicated no abnormalities. Organic acid profiling by gas chromatography and mass spectrophotometry indicated no abnormalities and no increase in lactate or methylmalonate. The glycosaminoglycan amount was 9.2 mg/mM cr.

## 4. Discussion

*LRRTM4* is a component of the neurexin trans-synaptic connexus (NTSC) involved in neuronal cell adhesion, synaptogenesis and neurotransmission (Figure 1) [7]. *LRRTM4* is part of a larger LRRTM family (*LRRTM1-4*) of postsynaptic ligands for the neurexins (NRXNs). Rare mutations of *NRXN1* are strongly associated with Tourette syndrome and autism. The other neurexins (*NRXN2-4*) are also associated with TS and ASD (Figure 1). Postsynaptic ligands of the NRXNs, including the NLGNs, the CBLNs and the LRRTMs, have all been associated with TS and ASD [7,14], but this is the first report of *LRRTM4* in association with TS and ASD.

TS and ASD more often affect males compared to females at a ratio of ~4:1. Notwithstanding, there is little evidence of strong male bias associated with *NRXN1* mutations in TS or ASD. In this study, we report two affected females from the same family in association with the duplication of the terminal exon of *LRRTM4*, suggesting that the male bias more generally associated with TS/ASD may not apply as strongly with the NTSC or *LRRTM4* association with TS/ASD.

*LRRTM4* is a paralog of *LRRTM3* which is also associated with TS and ASD [14]. *LRRTM3* overlaps the *CTNNA3* gene, which has recurrent partial deletions in TS and ASD [14]. *LRRTM3* is nested within and overlaps the *CTNNA3 gene*, where both genes are transcribed from opposing strands of the same DNA in convergent fashion [14]. We previously demonstrated that the transcription of *LRRTM3* is discordantly regulated by the convergent transcription of *CTNNA3*, thus extending the *CTNNA3* association with TS/ASD to include *LRRTM3* [14]. Likewise, *LRRTM1* overlaps and is nested within an intron of the *CTNNA2* gene and overlapping deletions have been identified near the 3′ end of *CTNNA2*, in a ~1Mb overlap region enriched with conserved DNA noncoding elements that may regulate the expression of *CTNNA2* and *LRRTM1* [26].

The duplication breakpoints identified in the Tourette syndrome family reported in this study were also localised near conserved non-coding regions on either side of the terminal exon of *LRRTM4* (Figure 2). Deletion of gene sequences has a more predictable impact on gene expression compared with duplications like the one reported in this study. Intragenic duplications may interfere with gene transcription or silence the gene, possibly by reversing the orientation of gene sequences or disruption of conserved gene regulatory elements that affect gene expression. In this study, it was not possible to test for *LRRTM4* gene expression in neuronal tissues; however, it is highly plausible that the partial duplication of *LRRTM4* may have affected the expression of *LRRTM4* in this family.

*LRRTM3* and *LRRTM4* are prominently expressed in the dentate gyrus [27]. Presynaptic NRXNs and type IIa receptor type protein tyrosine phosphatases organize synapses through a network of NTSC postsynaptic ligands including the LRRTMs. LRRTMs differentially engage the NRXNs through NRXN heparan sulfate modifications to induce presynaptic differentiation. Binding to the heparan sulfate modification of NRXN is sufficient for *LRRTM3* and *LRRTM4* to induce synaptogenesis. *LRRTM4* is the postsynaptic ligand for the potent synapse organiser *NRXN1δ*, and Roppongi et al. demonstrated that mice expressing a mutant form of *LRRTM4* that fails to bind the heparan sulfate modification of NRXN exhibit structural and functional deficits at dentate gyrus excitatory synapses [27]. Together these findings highlight the complex interplay of synapse organizers, including *LRRTM4*, in specifying the molecular algorithm for neuronal circuitry formation [27].

## Data Availability

Not applicable.
